# Blanching Pre-Treatment Promotes High Yields, Bioactive Compounds, Antioxidants, Enzyme Inactivation and Antibacterial Activity of ‘Wonderful’ Pomegranate Peel Extracts at Three Different Harvest Maturities

**DOI:** 10.3390/antiox10071119

**Published:** 2021-07-13

**Authors:** Tandokazi Pamela Magangana, Nokwanda P. Makunga, Chris la Grange, Maria A. Stander, Olaniyi Amos Fawole, Umezuruike Linus Opara

**Affiliations:** 1 Department of Botany and Zoology, Stellenbosch University, Private Bag X1, Matieland, Stellenbosch 7602, South Africa; tkmagangana@sun.ac.za (T.P.M.); makunga@sun.ac.za (N.P.M.); chrislag@sun.ac.za (C.l.G.); 2 SARChI Postharvest Technology Research Laboratory, Africa Institute for Postharvest Technology, Faculty of AgriSciences, Stellenbosch University, Private Bag X1, Stellenbosch 7602, South Africa; 3 Department of Biochemistry, Stellenbosch University, Private Bag X1, Matieland, Stellenbosch 7602, South Africa; lcms@sun.ac.za; 4 Postharvest Research Laboratory, Department of Botany and Plant Biotechnology, University of Johannesburg, P.O. Box 524, Auckland Park, Johannesburg 2006, South Africa; 5 UNESCO International Centre for Biotechnology, Nsukka 410001, Enugu State, Nigeria

**Keywords:** agriculture waste, flavonoids, ellagitannins, high performance liquid chromatography-photodiode array detection (HPLC-PDA), metabolite profiling, polyphenols, pomegranate fruit peel, value-addition

## Abstract

‘Wonderful’ pomegranate (*Punica granatum* L.) peel contains a wide range of phytochemicals including vitamins, dietary fibre, phenolic compounds, and antioxidant properties. Yet, it is often used as animal feed or discarded in landfills, which is not the best eco-friendly way to utilize this phenolic-rich bioresource. Finding novel ways of utilizing pomegranate peel waste could prove a more profitable and eco-friendlier alternative that is far more beneficial to the economy. Adding a blanching pre-treatment step at optimal conditions prior to processing of pomegranate peel aids in the inactivation of quality changing enzymes such as polyphenol oxidase (PPO) and peroxidase (POD), which are accountable for the degradation reactions that cause breakdown of nutrients and phytochemicals. This study aimed to determine the effect of blanching at 80 °C for 3 min on the yield, polyphenol content, antioxidant properties, enzyme inactivation, and antibacterial activity of ‘Wonderful’ pomegranate peel ethanolic extracts from three different harvest maturities (unripe, ripe, and over ripe), including a comprehensive characterization and quantification using liquid chromatography-mass spectrometry (LC-MS). The blanched unripe peel extracts exhibited the highest total phenolic content, total tannin content, 2,2-diphenyl-1-picryl hydrazyl (DPPH) antioxidant activity, 2,2-azino-bis (3-ethylbenzothiazoline-6-sulphonic acid (ABTS) radical scavenging activity and ferric ion reducing antioxidant power (FRAP) at 14.0 mg gallic acid equivalent (GAE)/g dry mass (DM), 1.0 mg GAE/g DM, 359.1 µmol Trolox/g DM, 912.2 µmol Trolox/g DM and 802.5 µmol Trolox/g DM, respectively. There was significant (*p* < 0.05) decrease in PPO and POD activity of all blanched pomegranate peel extracts. The blanched unripe peel extracts had the lowest PPO activity at 0.2 U/g fresh weight (FW), with a 70% PPO inactivation compared to ripe and over ripe harvest, whereas the highest POD inactivation was recorded at 67% in over ripe peel extracts. All blanched peel extracts, irrespective of harvest maturity, had minimum inhibitory concentration (MIC) values at 160 µg/mL against all four bacteria strains tested, which included two Gram-positive bacterial strains (*Bacillus subtilis* ATCC 6051 and *Staphylococcus aureus* ATCC 12600) and two Gram-negative bacteria (*Escherichia coli* 11775 and *Klebsiella pneumonia* ATCC 13883). A total of 25 metabolites including phenolic acids (4), organic acids (1), flavonoids (4), ellagitannins (13), and other polyphenols (3) in all three pomegranate peel samples were tentatively identified after LC-MS profiling. The blanched unripe peel extracts showed significantly higher punicalin α and β, β punicalagin, catechin, epicatechin content at 414 mg/g, and 678 mg/g, 151 mg/g, 229 mg/g, respectively, compared to peel extracts from other harvest maturities. This study provides supportive information for the commercial utilization of pomegranate fruit peel as source of value-added ingredients for the development of novel food, cosmetics, and pharmacological products.

## 1. Introduction

The pomegranate (*Punica granatum* L.; Lythraceae) is cultivated in many places around the world such as North America, South America, Asia, North Africa, Australia, Israel, the Middle East, and, most recently, South Africa [1,2,3]. Postharvest fruit loss and waste is a major problem in the pomegranate industry, especially on-farm [4] and packhouse level [5], mainly due to the presence of defects and disorders which reduce appearance quality. There are over a thousand cultivars world-wide but ‘Wonderful’ is the most widely cultivated pomegranate cultivar in South Africa due to its high quality and yields [6]. This particular cultivar has the highest cultivar distribution of 76% [7]. ‘Wonderful’ pomegranate also contributes to the majority of the pomegranate waste (in the form of seeds and peel) at an estimated 1406.49 tons at packhouse level in South Africa [7]. The peel has the highest phytochemical content and strong biological activity [3,8]. It is a good source of nutrients, dietary fibre, phenolic compounds which include flavonoids (anthocyanins and flavonols), phenolic acids (ellagic acid, gallic acid caffeic acids, ferulic acids), hydrolysable tannins (ellagitannins and gallotannins), and condensed tannins (proanthocyanadins), which are produced through shikimic acid metabolism [3,9,10,11,12]. These compounds vary in their chemical nature and play a significant role in the antimicrobial, anticancer, anti-cardiovascular, anti-inflammatory, and antioxidant activities [3,11,12,13,14]. The global demand for natural health promoting products presents an opportunity to turn pomegranate peel waste to commercial products.

Variations in chemical composition of these phenolic compounds in the fruit peel, however, are controlled by several factors, including cultivar, growing conditions, climate, cultural practices and harvest maturity [15,16]. For example, during the maturation of ‘Malas Yazdi’ pomegranate fruit, Mirdehghan and Rahemi [16] recorded an increase in total phenolic content (TPC) of the fruit peel in the early stages of maturation at almost 120 mg/g dry weight (DW), however, it declined to 50.2 mg/g DW at harvest maturity stage. Attanayake et al. [17] noted a gradual reduction in TPC from approximately 51 mg gallic acid equivalent (GAE)/g fresh weight (FW) to 30.6 mg GAE/g FW α punicalagin from approximately 11 mg/g FW to 9 mg/g FW and antioxidant activity from 75% to 41.3% during flowering stage to maturity of ‘Nimali’ pomegranate peel. Similarly, Li et al. [18] found that unripe maturity stage of Sweet green peel (GP) pomegranate peel recorded the highest TPC, approximately 390 mg GAE/g DW, compared to the ripe stage (250 mg GAE/g DW). The influence of harvest maturity on the chemical composition of pomegranate peel may directly affect the biological activity, especially the antioxidant activity associated with the use of the peel extracts in the downstream manufacturing pipeline.

During processes such as drying, freezing, and/or storage of peel, enzymatic reactions cause deterioration in the phytochemical and nutrient profiles reducing their content and quantity for value addition [19,20,21]. Effective methods of preserving these secondary metabolites and reducing their oxidation are crucial and still sought after. Hot water blanching is a pre-treatment step used prior to drying and aids in the inactivation of quality changing enzymes such as polyphenol oxidase (PPO) and peroxidase (POD). These enzymes lead to the degradation reactions that cause breakdown of nutrients and phytochemicals, off-flavours, odours, and undesirable texture and colour in vegetables and fruits [3,19,20,21]. The blanching technique is well recognised as a valuable and inexpensive method to inactivate browning enzymes such as PPO and POD, increasing extractability by loosening cellulosic structures within the peel [3,19,20,22,23]. Blanching also assists in reducing prolonged drying time of the peel, therefore preserving the integrity of the compounds during thermal processing [3,20,22]. On the other hand, over blanching may lead to the loss of water-soluble polyphenols by leaching [22,23,24]. Blanching is a practical step to produce high-quality value-added products from peel waste but there is no single method that is being applied routinely across different species and cultivars. Its effects may depend on the type and size of plant material, thermal stability of different phytochemicals, location of phytochemicals within the plant structure, enzyme activity, and cultivar differences [3,19,20,21,23,25].

To the best of our knowledge, no blanching pre-treatment technique has been used to assess the effect of blanching on the chemical composition, enzyme inactivation, and antibacterial activity of ‘Wonderful’ peel extracts from three different harvest maturities (unripe, ripe, and over ripe). Extraction of natural products focusing on the novel processes with reduced energy consumption, and renewable natural product is in demand in the industry, and in other work blanching has been shown to reduce the time needed for drying of plant materials prior to extraction [26]. The foundation of our hypothesis was based on the idea that blanching may inactivate or lower the activity of enzymes PPO and POD, and thus aid in higher extraction of commercially valuable polyphenols. We also hypothesized that blanching effect may vary according to the developmental stage (unripe, ripe, and over ripe) of pomegranate peel extracts and therefore affect the phytochemical profile of peel extracts positively at each developmental stage, which in turn will improve the bioactivity of the blanched peel extracts. Based on our previous study [27], we blanched at 80 °C for 3 min using ‘Wonderful’ at three harvest maturities, and with the aim of better defining the phytochemical effects of blanching, we determined the polyphenolic content and mapped their antioxidant potential using a variety of different in vitro spectrophotometric based assays to test this hypothesis. We further determined the enzyme activity of PPO and POD, and evaluated the effect of blanching on several commercially valuable compounds using a liquid chromatography mass spectrometry-based profiling technique. Furthermore, we evaluated the antibacterial effect of blanched peel extracts against two Gram-positive (*Bacillus subtilis* ATCC 6051 and *Staphylococcus aureus* ATCC 12600) and two Gram-negative (*Escherichia coli* 11775 and *Klebsiella pneumonia* ATCC 13883) bacteria.

## 2. Materials and Methods

### 2.1. Chemicals and Reagents

Catechin, ellagic acid, epicatechin, gallic acid, punicalagin α, punicalagin β, punicalin α and β, chlorogenic acid, rutin, syringic acid, quercetin, high performance liquid chromatography (HPLC)-grade methanol, formic acid, and acetonitrile were used for phenolic compound analysis. Other reagents included Folin-Ciocalteu, 2,2-diphenyl-1-picryl hydrazyl, 2,4,6-tri[2-pyridyl]-s-triazine (TPTZ), 2,2-azino-bis (3-ethylbenzothiazoline-6-sulphonic acid), ferric oxide and L-ascorbic acid (referred to here also as Vitamin C). All chemicals and reagents were purchased from Sigma-Aldrich, Darmstadt, Germany.

### 2.2. Plant Material

Pomegranate fruit (‘Wonderful’) were harvested at different harvest maturity stages (unripe, ripe, and over ripe) from Blydeverwacht farm, Wellington (33°48′0″ S, 19°53′0″ E) in the Western Cape Province, South Africa. Sampled pomegranate trees were between 5 and 7 years and used a drip irrigation system. Healthy fruit per orchard were harvested on the 15 March, 29 March, and 12 April 2019 for unripe, ripe, and over ripe pomegranate fruit, respectively. The fruit were transported to the Postharvest Technology at Stellenbosch University (Stellenbosch, South Africa) in an air-conditioned vehicle and stored at 7.5 ± 0.5 °C, 95% relative humidity (RH) before processing.

### 2.3. Sample Preparation and Blanching Procedure

Clean pomegranate peels were collected after juice extraction using a hand juice pressing machine. The peels were cut in dimensions of 20 ± 0.5 mm for both length and width and 5 ± 0.5 mm for thickness. The peels were blanched in a water bath (model 102, Scientific Engineering (Pty) Ltd., Maraisburg, South Africa) at 80 ± 2 °C for 3 min. This condition was based on a previous study from our laboratories [27]. Blanched peels were quickly soaked in cool water for 30 s to stop the blanching process and carefully drained. Unless otherwise stated, the blanched peels were then oven dried at 60 ± 2 °C at a relative humidity of 18.6% and 1.0 m/s air velocity for 16 h to reach a moisture content of 8% (*w*/*w*) [28]. The unblanched peel were taken as the control and this experiment was performed in triplicate. The moisture content of the peels was determined using moisture analyzer (DBS60-3, KERN, Balingen, Germany) at 100 °C. The dried peels were ground to a particle size less than 1 mm and stored −20 °C until extraction [29].

### 2.4. Phytochemical Extraction

Ultrasound assisted solvent extraction was used for extraction from the ground (particle size less than 1 mm) samples according to Wang et al. [28] with minor modifications, using an ultrasonic bath (model 705, Scientific Engineering, Maraisburg, South Africa) with maximum power of 700 W, 40 kHz frequency and internal dimensions (500 mm × 300 mm × 150 mm). The ultrasound extraction was carried out according to the following experimental conditions: maximum power of 40 kHz and 700 W, temperature of 40 ± 2 °C, time of 1 h, and solid-solid ratio of 15:1 (*w*/*v*). Dried peel powder (10 g) was mixed with 70% (*v*/*v*) ethanol.

### 2.5. Determination of Phytochemicals and Antioxidant Activity

#### 2.5.1. Extract Yield

Dried peel powder (10 g) was mixed with 70% (*v*/*v*) ethanol, sonicated, and filtered with Whatman filter paper number 1 under vacuum. The extracts were further air-dried in a fume hood at room temperature. Afterwards, vacuum evaporation (G3, Heidolph, Schwabach, Germany) at 50 ± 2 °C was used to remove any solvent residue. All the tests were run in triplicate. Dried extracts were weighed to calculate extract yield according to Equation (1) below:

Total extract yield (%) = g dried extract/100 g peel powder × 100(1)

The extract yield is expressed as % per g DM.

#### 2.5.2. Total Phenolic Content (TPC)

The TPC of extracts from pomegranate peels was determined using the Folin-Ciocalteu reagent (Folin-C) according to a method described by Fawole et al. [8] with minor modifications. Sample extract (50 µL) was mixed with 450 µL of 50% methanol and afterwards 500 µL Folin C was added to the mixture. After 2 min, 2.5 mL of 2% sodium carbonate was added to the mixture, vortexed for 30 s followed by an incubation step in the dark at room temperature for 40 min. Afterwards, the absorbance was measured at 725 nm using a UV-visible spectrophotometer (Thermo Scientific Technologies, Madison, WI, USA). Each sample was tested in triplicate and quantification was based on a standard curve that was generated with 0–0.014 mg/mL gallic acid ethanolic solution. Results are expressed in mg gallic acid equivalents (GAE) per g dry mass.

#### 2.5.3. Total Tannin Content (TTC)

Total tannin content (TTC) of extracts of pomegranate peels was based on a procedure determined by Makkar [30]. Briefly, for extraction of tannins, 100 mg of polyvinylpolypyrrolidone (PVPP) was added to 1 mL of distilled water and 1 mL peel extracts. Thereafter, the mixture was vortexed for 30 s and kept for 15 min at 4 °C, before it was centrifuged at 4000× *g* for 10 min. Thereafter, 50 µL of the supernatant was dissolved in 50% methanol, followed by the addition of 500 µL of Folin-C and then 2.5 mL of 2% sodium carbonate after 2 min. Afterwards, the mixture was incubated for 40 min at room temperature in the dark. After incubation, the absorbance was recorded using a UV-visible spectrophotometer (Thermo Scientific Technologies, Madison, WI, USA) at 725 nm. Separate peel extracts not treated with PVPP were measured for total phenolic content. The TTC calculation was based on Equation (2) below:

TTC = TPC _(in peel extract without PVPP)_ − TPC _(in peel extract treated with PVPP)_ (2)

#### 2.5.4. Total Flavonoid Content (TFC)

The method of Yang et al. [31] was used for the total flavonoid content (TFC) of pomegranate peels. Briefly, peel samples (0.01 g) were extracted using 50% methanol (10 mL) and vortexed for 30 s and then sonicated for 10 min in an ultrasonic bath. Afterwards, the mixture was centrifuged at 4000× *g* for 12 min at 4 °C. After that, 1.2 mL of distilled water was added to 250 µL of extracted peel extracts and then followed by an addition of 75 µL of 5% sodium nitrite. After 6 min, 150 µL of 10% aluminium chloride (AlCl_3_) and 500 µL of 1 mM sodium hydroxide were added to the mixture. Finally, the mixture was adjusted to 3 mL with distilled water and further vortexed for 30 s before absorbances were recorded at 510, using a using a spectrophotometer (Thermo Scientific Technologies, Madison, WI, USA), for each sample. The TFC is expressed as mg catechin equivalents per g peel extracts through the calibration curve with catechin. The calibration curve range was 0–0.5 µg/mL. Triplicate measurements were taken for all samples.

#### 2.5.5. Total Anthocyanin Content (TAC)

For the TAC measurements, the protocol of Wrolstad [32] was used and this involved using 1 mL of the peel extract being mixed with 9 mL of pH buffers of 1.0 and 4.5, respectively. Thereafter, the absorbance was recorded spectrophometrically (Thermo Scientific Technologies, Madison, WI, USA) at 520 nm and 700 nm for each of the two buffers, respectively. Each sample was tested in triplicate. The results are expressed as cyanidin 3-glucoside equivalent (C_3_gE) per g dry matter (mg C3gE/g DM) and Equations (3) and (4) (shown below) were used to calculate the total absorbance and total anthocyanin content, respectively. The final results are reported as:

A= (A510 − A700)_pH1.0_ − (A510 − A700)_pH4.5_ (3)

(4) $$\mathrm{Total}\;\mathrm{anthocyanin}\;\left(µg/\mathrm{mL}\right)=\frac{\left(A\times \;\mathrm{MW}\;\times \;\mathrm{DF}\right)}{\varepsilon \;\times \;L}$$

where A = Absorbance, ε = Cyd-3-glucoside molar absorbance (26,900), MW = anthocyanin molecular weight of 449.2, DF = dilution factor, L = cell path-length (1.0 cm).

#### 2.5.6. Ascorbic Acid Content (Vitamin C)

The assay was carried out as described by Mphahlele et al. [13]. Peel extracts (1 g) were mixed with 1% metaphosphoric acid (50 mL) and then sonicated in ice for 4 min and centrifuged at 4000× *g* for 12 min. The supernatant of 1.0 mL was pipetted into a tube and mixed with 9 mL of 2,6 dichlorophenolindophenol dye (0.0025%). Afterwards, the mixture was incubated at room temperature in the dark for 10 min before absorbance readings being taken at 515 nm. A calibration curve (0.01–0.1 µg/mL) using an authentic L-ascorbic acid chemical standard was used to calculate the ascorbic acid concentration, using triplicates. The results are expressed as ascorbic acid equivalents per g dry matter (µg of AAE/g DM).

#### 2.5.7. 2,2-Diphenyl-1-picryl Hydrazyl (DPPH) Antioxidant Assay

The radical scavenging activity of pomegranate peels was measured by DPPH assay based on the method of Karioti et al. [33], with minor modifications as described by Fawole et al. [8]. Peel extracts of 15 µL were mixed with 735 µL methanol and 0.1 mM solution of DPPH (750 µM) and incubated for 30 min in the dark at 25 °C. After incubation, the absorbance was measured at 517 nm using a UV-visible spectrophotometer (Thermo Scientific Technologies, Madison, Wisconsin). Sample quantification was based on the standard curve of 0–1500 μM and each sample was tested in triplicate. The results are recorded as µmol Trolox/g DM according to Equation (5), as shown below:

RSA % = [1 − (A_test_/A_blank_) × 100] (5)

where A_test_ represents the absorbance of the reaction mixture which has the standard or extract, and A_blank_ represents the absorbance of the blank test.

#### 2.5.8. Ferric Ion Reducing Antioxidant Power (FRAP) Antioxidant Assay

The ferric reducing capability of the pomegranate peels was carried out using the method described by Benzie and Strain [34], with some modifications by Fawole et al. [8]. The ferric ion reducing antioxidant power (FRAP) reagent was made fresh daily by mixing 25 mL of acetate buffer (300 mM acetate buffer at pH 3.6), 2.5 mL (10 mM of 2,4,6-tri[2-pyridyl]-s-triazine (TPTZ) solution) and 2.5 mL (20 mM ferric chloride (FeCl_3_) solution). Ten mililitres of 50% methanol was mixed with 1 mL peel extract, sonicated in cold water for 10 min and centrifuged at 4 °C for 5 min. Each peel extract (150 µL) was added to 2850 µL FRAP, vortexed for 30 s and incubated for 30 min in the dark at 25 °C. After incubation, absorbance was taken at 593 nm using a UV-vis spectrophotometer (Thermo Scientific Technologies, Madison, WI, USA). Each sample was tested in triplicate and the results are stated as µmol Trolox/g DM.

#### 2.5.9. 2,2-Azino-Bis (3-Ethylbenzothiazoline-6-sulphonic Acid) (ABTS) Antioxidant Assay

The ABTS + radical cation decolourization assay, as described by Chirinos et al. [35], was used to measure the antioxidant activity of different peel extracts, using triplicates. Briefly, potassium persulfate solution was freshly prepared at 2.6 mM and mixed with 7.4 mM ABTS solution (1:1, *v*/*v*) and then incubated in the dark at 25 °C for 12 h to generate an ABTS^+^ stock solution. The ABTS+ solution was further diluted with methanol to gain absorbance of 0.70 ± 0.02 at 750 nm. A 15 µL peel extract was added to 200 µL of the ABTS working solution. The reaction was allowed to proceed for 6 min and all measurements of the absorbance were recorded at 750 nm to generate values of the Trolox equivalents per g sample (µmol Trolox/g DM).

### 2.6. Enzyme Activity Evaluation

#### 2.6.1. Sample Preparation

In order to investigate the activity of enzymes on pomegranate peel after blanching, plant materials were not subjected to drying but ground to a fine powder using a pestle and mortar with liquid nitrogen. One gram of blanched pomegranate peel powder was diluted in 10 mL cold extraction buffer (0.1 M phosphate buffer at pH 7, 0.05 M/L EDTA and 60 g/L polyvinyl polypyrrolidone (ratio of 1:1:1)). Then, the samples were vortexed for 30 s, sonicated in cold water for 10 min, and stored in the dark at 4 °C for 2 h. After incubation, the samples were centrifuged at 4000 rpm for 25 min and the resultant supernatant were taken into clean vials and stored at −80 °C for further analysis. The same procedure was performed also for the fresh unblanched peel samples.

#### 2.6.2. Polyphenol Oxidase (PPO) Assay

Polyphenol oxidase activity was determined according to the method of Gonzalez et al. [36] with minor modifications indicated by Arendse et al. [37]. The activity of the PPO enzyme was measured by increasing the rate of absorbance at 420 nm using UV-vis spectrophotometer (Thermo Scientific Technologies, Madison, WI, USA) for 3 min. The activity was assayed in a 3 mL reaction mixture containing the enzyme extract of 0.2 mL and followed by the addition of 0.3 mL (0.1 M) catechol and 2.5 mL of potassium phosphate buffer (0.1 M, pH 6). The activity was expressed as change in absorbance at 420 nm over a 3 min period. The blank consisted of 3 mL sodium phosphate buffer at pH 6 and the results are expressed as unit per g of fresh weight (U/g FW).

#### 2.6.3. Peroxidase (POD) Assay

For the peroxidase activity assay, the method of Meighani et al. [38] with slight modifications by Arendse et al. [37] was followed. The reaction mixture contained 2.73 mL of sodium phosphate buffer (0.1 M, pH 6), 0.1 mL of guaiacol (0.045 M), 0.15 mL of hydrogen peroxide, and 0.02 mL enzyme extract. The reaction started when 0.1 mL of guaiacol substrate was added. The activity was expressed as change in absorbance using a UV-vis spectrophotometer (Thermo Scientific Technologies, Madison, WI, USA) at 470 nm over a 2 min period. The results are recorded as unit per g fresh weight (U/g FW).

### 2.7. Ultra-Liquid Chromatography Mass Spectrometry

A method described by Mphahlele et al. [13] was used for these analyses. All samples were analysed using a Waters Synapt G2 quadrupole time-of-flight mass spectrometer (Milford, MA, USA). The instrument was connected to a Waters Acquity ultra-performance liquid chromatograph (UPLC) and Acquity photo diode array (PDA) detector. Ionisation was achieved with an electrospray source using a cone voltage of 15 V and capillary voltage of 2.5 kV. Nitrogen was used as the desolvation gas at 650 L/h and the desolvation temperature was set to 275 °C. The separations were carried out on a Waters HSS T3 column (2.1 × 150 mm, 1.8 μm particle size), with an injection volume of 3 μL at a flow rate of 0.25 mL/min (operated at 20 °C). The mobile phase contained solvent A (0.1% formic acid) and solvent B (0.1% acetonitrile), and the initial composition was 100% A and 0% B. A gradient elution was employed where phase B increased linearly from 0% to 5% over the first 1.10 min, then increased to 25% over 17 min, and finally 100% at 18 min. The column was subjected to 100% solvent B for an extra 3 min. The column was then re-equilibrated over 5 min to yield a total run time of 26 min. Reference standards of phenolic acids and flavonoids (Sigma-Aldrich, Darmstadt, Germany) were used for the quantification of individual compounds in pomegranate peel extracts (described directly below). For these analyses, five replicates were used for each sample using a scanning range of *m/z* 120 to 1500.

For compound identification, the mass spectrometry ramp collision energy MS^E^ scanning mode was utilized to acquire both molecular and fragment ionization data, where data were acquired using two channels. The first was a low collision energy at 4 V followed by a high collision energy ramped from 20 to 60 V. This was done to obtain molecular ionisation and fragment data to enable tentative assignments of some of the chemicals that are currently presented in this study, and the tentative identities were further compared to published results. Electron spray ionisation in negative mode was achieved for a scanning range of 120 to 1500 *m/z*.

#### 2.7.1. Determination of Individual Phenolic Acids and Flavonoid Concentration

The measurement and structural confirmation of some of the metabolites was possible with pure standards (catechin; ellagic acid; epicatechin; gallic acid; punicalagin α; punicalagin β; punicalin α and β; chlorogenic acid; rutin; syringic acid; and, quercetin; Sigma-Aldrich, Darmstadt, Germany). The standards were dissolved in methanol as cocktails to a concentration range of 0.1–50 µg/mL and were used as external calibrants for both quantitative and qualitative analysis. In fact, the extracted mass chromatogram of the molecular ion of the respective compound was used for quantification.

#### 2.7.2. Data Acquisition

For data acquisition and processing, MassLynx 4.1 software (Central Analytical Facilities (CAF), Stellenbosch, South Africa) was utilized. A metabolomics approach was used to reveal major and subtle differences and similarities (variance) between the samples. Twenty-five metabolites were thus annotated. Compounds (catechin, epicatechin, gallic acid, ellagic acid, α punicalagin, β punicalagin, and punicalin α and β) were quantified using univariate statistics.

### 2.8. Microdilution Antimicrobial Assay

A method of Eloff [39] with slight modifications by Fawole et al. [8] was used to test the antibacterial activity of blanched pomegranate peel extracts ‘Wonderful’ from three different harvest maturities (unripe, ripe, and over ripe). In brief, activity was tested against two Gram-positive bacterial strains (*Bacillus subtilis* ATCC 6051 and *Staphylococcus aureus* ATCC 12600) and two Gram-negative bacteria (*Escherichia coli* 11775 and *Klebsiella pneumonia* ATCC 13883). Cultures were grown overnight in sterile Mueller-Hinton (MH) broth. The initial concentration of 10 mg/mL of peel sample was prepared by dissolving dried extracts in 70% (*v*/*v*) ethanol. Extracts (100 µL per well) were serially diluted with sterile distilled water in a 96-well-microplate and a 0.5 McFarland absorbance at 600 nm was used as a reference. This was done in triplicate. The bacterial suspension (100 µL) was added to each well. One positive antibiotic control (0.1 mg/mL Streptomycin; Sigma-Aldrich, Saint Louis, MO, USA) and two negative controls (bacteria-free broth, 70% (*v*/*v*) ethanol and sterile distilled water) were set up against each bacterium. The final concentration of peel extracts ranged from 19.5 to 2500 µg/mL, and streptomycin concentrations ranged from 0.20 to 25 µg/mL, in each respective wells. The microplates were incubated at 37 °C for 18 h before 40 µL of *p*-iodonitrotetrazolium chloride (INT; Sigma-Aldrich, Darmstadt, Germany) was added to each well. These plates were further incubated for 1 h and were examined thereafter. Bacterial growth/survival was clearly visible as these wells turned pink. The clear wells indicated the inhibition of bacterial growth and thus the minimum inhibitory concentration (MIC) value was recorded as the lowest concentration that could inhibit bacterial growth.

### 2.9. Statistical Analyses

Normality testing was conducted before analysis of variance (ANOVA) and then all data that had a normal distribution were then subjected to a post-hoc analysis according to Duncan’s multiple range test to separate the means. In cases where the normal assumptions were not followed, the Kruskal-Wallis was used as a post-hoc test. The statistical significance was calculated using SAS Software (SAS Enterprise Guideline 7.1, SAS Enterprise, Carrey, NC, USA) and the results of all the studied variables are presented as mean (±S.E); (*p ≤* 0.05). These tests were applied for the phytochemical and antioxidant activities; and, the quantitative measurements of the biomarker phenolics.

The metabolomic data were normalized by using pooled averages and for data-scaling, the auto-scaling algorithm was used prior to multivariate statistical analyses. A heat map, two-dimensional (2D) principal component analysis (PCA) score plot and a PCA biplot were generated. In addition, a biplot showing phenolic compound distribution and concentrations between the harvest maturities (unripe, ripe, and over ripe) of ‘Wonderful’ pomegranate peel extracts was also made. All the above plots were constructed using MetaboAnalyst 5.0 (a web-based metabolomics interface hosted by the Wishart Group, Alberta, Canada) (www.metaboanalyst.ca (accessed on the 19 February 2021)). Finally, a Pearson’s correlation analysis was done using XLSTAT software (version 2019.4.1.63305, Addinsoft, New York City, USA), which included the phytochemical colourimetric analyses, antioxidant enzyme, and antibacterial assays plus biomarker phenolic metabolites in the data matrix.

## 3. Results

### 3.1. Polyphenol Analysis (Extract Yield, TPC, TTC, TFC, TAC, and Vitamin C)

The pomegranate fruit peel has substantial amounts of potentially valuable components such as phenolic acids, flavonoids, and ellagitannins [3,8,11]. Our aim was to determine the effects of blanching on yield, bioactive compounds, antioxidant, enzyme inactivation, and antibacterial activity of ‘Wonderful’ pomegranate peel extracts at three different harvest (unripe, ripe, and over ripe) maturities. We demonstrate for the first time that blanching at 80 °C for 3 min of pomegranate peel significantly (*p* < 0.05) increased the extract yield of ‘Wonderful’ pomegranate peel at different harvest maturities (Table 1). However, the degree of blanching improvement on the quality attributes tested varied on the harvest maturity status (unripe, ripe, and over ripe harvest) of ‘Wonderful’ pomegranate peel extracts level. For instance, the extractability of polyphenols in unripe peel was greatly improved by blanching compared to the ripe and over ripe stage. Blanched unripe peel extracts had the highest TPC, TTC, and TAC, at 14.0 ± 0.0 mg GAE/g DM, 1.0 ± 0.1 mg GAE/g DM, and 0.1 ± 0.0 mg Cy3dE/g DM, respectively. The polyphenol content declined significantly with advancing maturity, whereas the contents of TFC and vitamin C increased significantly (*p* < 0.05) with advancing maturity. Furthermore, vitamin C reduced significantly (*p* < 0.05) when blanching was applied at unripe and over ripe harvest maturities except at ripe harvest stage, where blanching increased the vitamin C concentration.

### 3.2. Antioxidant Activity (DPPH, FRAP, and ABTS)

Antioxidant assays which include 2,2 diphenyl-1-picryl hydrazyl assay (DPPH), and 2,2-azino-bis(3-ethylbenzothiazoline-6-sulphonic acid assay) (ABTS) were used to measure the radical scavenging ability, while ferric reducing antioxidant power assay (FRAP) was used to determine the reducing power of the peel samples. The antioxidant activities of pomegranate peel extracts at different stages of fruit maturation are shown in Table 1. Total antioxidant activity followed the same trend as phytochemical content, and there was a significant decrease in the antioxidant activity (DPPH, ABTS, FRAP assays) in ‘Wonderful’ pomegranate peel extracts with advancing maturity. Moreover, the blanched peel extracts from all harvest maturities were recorded and the highest antioxidant potential was compared to unblanched peel extracts obtained from the three different harvest maturities (Table 1). The highest antioxidant potential was at the unripe stage, at 359.2 ± 0.0 µmol Trolox/g DM, 912.2 ± 0.0 µmol Trolox/g DM, and 802.5 ± 4.3 µmol Trolox/g DM, for DPPH, ABTS, and FRAP assays, respectively. Moreover, differences in the phenolic structural profiles of the extracts led to changes in the antioxidant potential for the extracts (Figure S1). This was largely due to variances in harvest maturity and blanching treatment used in this study, where peel extracts from the unripe stage showed stronger antioxidant potential. All blanched peel extracts regardless of harvest maturity stage accumulated higher polyphenols as a result of blanching treatment except for β-punicalagin and epicatechin in the unripe and over ripe stage, respectively (Table 2). Blanched pomegranate extracts from the unripe stage had significantly higher catechin and epicatechin at 151 ± 6 mg/g, and 22 ± 1 mg/g (*p* < 0.05), respectively, whereas over ripe peel extracts had significantly (*p* < 0.05) higher α-punicalagin and gallic acid at 659 ± 19 mg/g and 200 ± 9 mg/g, respectively. These data lend support to the higher antioxidant activities reported in Table 1.

### 3.3. Enzyme Activity (PPO and POD)

Enzyme activity is regarded as one of the most important factors responsible for producing chemical changes in phytochemicals [20,40]. As shown in Table 1, there was a significant (*p* < 0.05) decrease in PPO and POD activity from all blanched pomegranate peel extracts. Enzyme activity followed the same trend as the phytochemical content and antioxidant activity. Blanched ‘Wonderful’ pomegranate peel extracts at unripe maturity stage were reported with the lowest PPO activity at 0.2 ± 0.1 U/g FW, with a 70% PPO inactivation compared to ripe and over ripe harvest at 17.5% and 17.2%, respectively. Similarly, POD activity declined significantly in blanched peel extracts with a 60%, 50% and 67% POD inactivation for unripe, ripe, and over ripe harvest, respectively. However, complete inactivation of PPO and POD was not attained. Enzymes such as PPO and POD contribute to the oxidation of phenolic compounds leading to the degradation of valuable phenolics [19,20,21,40].

### 3.4. Antibacterial Activity

The antibacterial activity of unblanched and blanched ‘Wonderful’ pomegranate peel extracts from three harvest maturities (unripe, ripe, and over ripe) is presented in Table 3. All ethanolic extracts showed good to moderate antibacterial activity with all extracts exhibiting MIC values between 160 µg/mL and 310 µg/mL, irrespective of bacterial strains tested. Furthermore, there was no particular trend connecting the antibacterial activities with any particular maturity stage. Although unripe peel extracts had significantly higher antioxidant activity compared to other harvest maturities, all blanched peel extracts showed consistency in their relatively higher antioxidant and antibacterial activities. All blanched peel extracts were two times lower in their MIC value at 160 µg/mL than the unblanched peel extracts against all four bacteria strains tested regardless of the harvest maturity of the pomegranate peel extracts.

### 3.5. Correlation Matrix and Principal Component Analysis

The Pearson’s correlation was used to reveal the degree of correlation between selected reference data and variables (Table 4). As expected, TPC was positively correlated to TTC and TFC at r = 0.84, *p =* 0.05 and r = 0.83, *p =* 0.05, respectively. Significant positive correlations were recorded for DPPH and TPC (r = 0.94, *p =* 0.05), and between FRAP and TAC (r = 0.97, *p =* 0.05). PPO showed a significant (*p* = 0.05) high negative relationship with TAC and FRAP (r = −0.90), respectively, suggesting that the decrease in PPO activity as a result of blanching contributes to higher TAC and FRAP antioxidant activity. Moreover, strong negative correlation is indicated between POD and extract yield (r = −0.86, *p =* 0.05). DPPH and α-punicalagin (α-PC) were reported with strong positive correlation (r= 0.85, *p =* 0.05), whereas DPPH had strong negative correlation with all bacteria strains tested (r = −0.87, *p =* 0.05) suggesting α-PC may be responsible for the high DPPH antioxidant potential. Therefore, contributing to the decrease in all bacterial strains tested. This is in agreement with our results, which showed that lowered activity of PPO and POD enzymes during blanching leads to high recovery of phytochemicals and therefore higher antioxidant and antibacterial activity.

**Figure 1:**
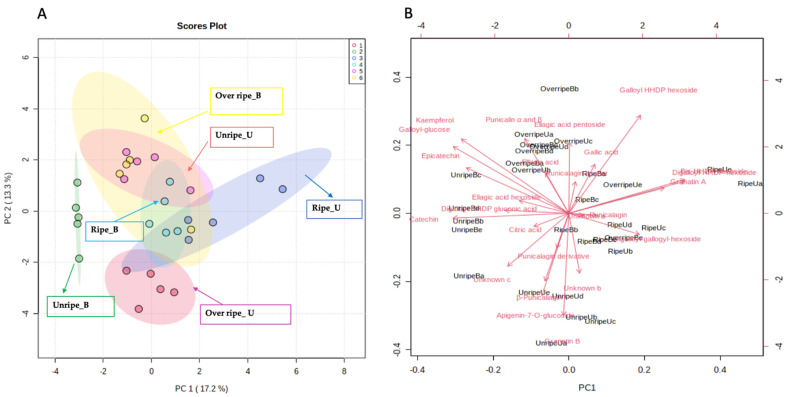
(**A**) Principal component analysis (PCA) score plot and (**B**) Principal component analysis (PCA) biplot showing the potential biomarkers linked to the three different harvest maturities (unripe, ripe, and over ripe) of ‘Wonderful’ pomegranate peel extracts blanched at 80 °C for 3 min. Letters U and B represent unblanched and blanched pomegranate peel extracts at 80 °C for 3 min.

A two-dimensional (2D) principal component analysis (PCA) score plot and PCA biplot were generated to demonstrate distinctness between harvest maturities and blanching treatments tested based on their LC-MS metabolite profiles (Figure 1A). The score plot was conducted between components 1 and 2, accounting for 30.5% of the variance of the data set. From the score plot (Figure 1A), six detectable groupings that are evident, which include unripe_U, unripe _B, ripe _U, ripe _B, over ripe_U and over ripe_B, made up of untreated unripe peel extracts; blanched unripe peel extracts; untreated ripe peel extracts; blanched ripe peel extracts; untreated over ripe peel extracts and blanched over ripe peel extracts, respectively. To identify the features responsible for the clustering patterns illustrated on the score plot, a biplot (Figure 1B) based on the PCA shows which features (shown as vector arrows specifying the accurate compound or mass/retention time) are responsible for the differences in the spatial distribution observed in the PCA score plot. The first cluster is composed of samples produced from the unripe blanched extracts, which had remarkable high phenolic content and antioxidant activities, as detailed in Table 1. These blanched unripe peel extracts were reported with the highest TPC, TTC, DPPH, FRAP and ABTS at 14.0 mg GAE/g DM, 1.0 mg GAE/g DM, 359.2 µmol Trolox/g DM, 802.5 µmol Trolox/g DM, 912.2 ± 0.0 µmol Trolox/g DM, respectively. The compounds associated with this cluster (unripe_B) are flavanols (catechin and epicatechin) (refer to the Table 5 for details).

**Figure 2:**
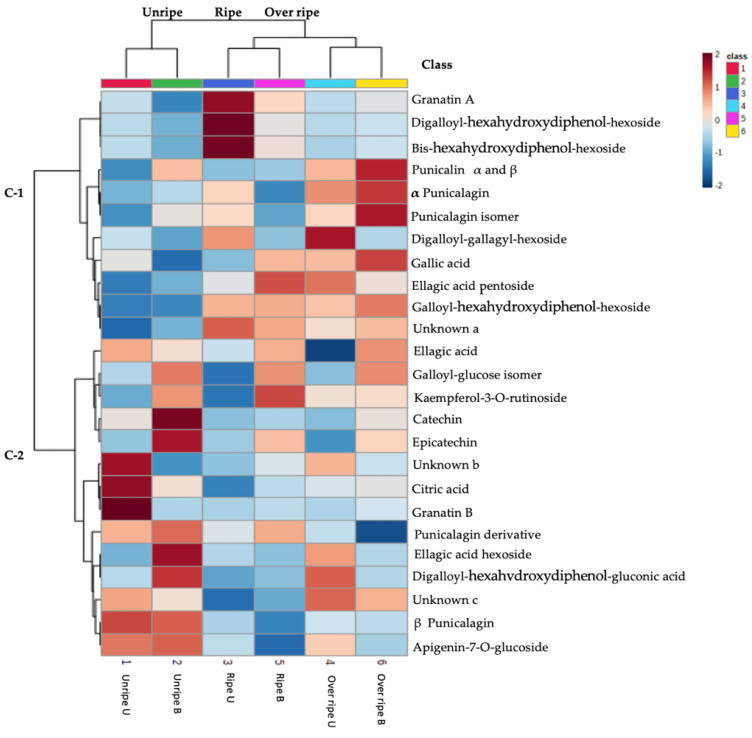
Heat map showing phenolic compound distribution and concentration between the harvest maturities (unripe, ripe, and over ripe) of ‘Wonderful’ pomegranate peel extracts. The red boxes mean concentrations are higher among different pomegranate fruit samples. The blue boxes show lower concentrations. Unripe, ripe, and over ripe harvested two weeks before commercial harvest, at commercial harvest and two weeks after commercial harvest, respectively. Letters U and B represent unblanched and blanched pomegranate peel extracts at 80 °C for 3 min, and C represents the cluster(s).

Blanching at 80 °C for 3 min contributed greatly to improved extraction of these polyphenols in unripe peel extracts, which could be a result of reducing enzymatic (PPO and POD) activity responsible for the degradation of polyphenols, and loosening cellulosic structures in the peel, resulting in high extractability of phytochemicals [22]. Phenolic contents were also grouped into two main clusters (C1 and C2), and the dendrogram were further grouped into different sub-clusters according to the similarities of their concentration patterns in the pomegranate peel samples (Figure S2). Overall, C1 to C2 clusters indicated several ellagitannins (granatin B, digalloyl-HHDP-hexoside (pedunculagin II), galloyl glucose isomer, β punicalagin and punicalagin derivative) phenolic acids (ellagic acid, and ellagic acid hexoside), flavonoids (catechin, epicatechin, kaempferol-3-O-rutinoside and apigenin-7-O-glucoside), organic acid (citric acid), as well as two unknowns (b and c) with [M-H]^−^ at *m/z* 353.0731 and 539.2146, at retention times of 12.37 and 12.81, respectively. These compounds had greater similarities in terms of the concentration among different pomegranate peel samples (Figure 2).

The largest peaks, presented in Figure S1, were used to characterize the profiles of the major chemicals present in ‘Wonderful’ pomegranate peel extracts across all harvest maturities (Table 5). A total of 25 compounds were tentatively identified by the interpretation of their fragmentation patterns obtained from mass spectra. Extracts of peel ‘Wonderful’ are complex with high variation quantitatively in their pool of compounds with antioxidant power. Chemical structures of some phenolic acids, flavonoids, and ellagitannins identified are presented (Figure S3). In the present study, there were several phenolic acids, flavonoids, and ellagitannins that were recorded and were strongly correlated to antioxidant and antimicrobial activity. Two monoglycosylated ellagic acid derivatives were tentatively characterized as ellagic acid-hexoside, and ellagic acid pentoside with a molecular formula of C_20_H_15_O_13_, and C_19_H_13_O_12_, respectively, and these have precursor ions at *m/z* 463.0539, and 433.0307. Ellagic acid pentoside was previously detected in selected Tunisian pomegranate peels, namely ‘Acide’, ‘Gabsi’, ‘Nebli’ and ‘Tounsi’ [44], while ellagic acid hexoside was previously characterized in peel fractions of pomegranate from USA [41]. Apart from the phenolic acids described above, several flavonoid conjugates (namely flavanols, flavones, and flavonols), were found in all pomegranate peel extracts of ‘Wonderful’ such as catechin, epicatechin, flavanol derivatives, apigenin-7-O-glucoside, and kaempferol-3-O-rutinoside.

## 4. Discussion

The unripe peel extracts generally had higher levels of the phytochemical content and antioxidant activity compared to other harvest maturities and decreased with advancing maturity (Table 1). In most fruits, harvest maturity stage markedly affects the quantity of the phenolic compounds [8,18]. The degradation of some phenolic compounds may be faster or slower than the biosynthesis of other phenolic compounds. According to other studies, phenolic acid concentrations decreased during maturity, whereas flavonoid concentrations increased during ripening [49]. In this study, it has been shown that there are both qualitative and quantitative differences in the concentrations of secondary metabolites in different harvest maturities strongly influenced by ripening and blanching treatments. Blanched unripe peel extracts had significantly (*p* < 0.05) higher catechin and epicatechin at 151 ± 6 mg/g, and 22 ± 1 mg/g, respectively, whereas over ripe peel extracts had significantly (*p* < 0.05) higher α-punicalagin and gallic acid at 659 ± 19 mg/g and 200 ± 9 mg/g, respectively. The variation of polyphenols depends on many factors, such as the cultivar, maturity stage, processing conditions, and environmental conditions during fruit development [3,13]. Our results are in line with that of the authors Mirdehghan and Rahemi [16] who observed an increase in total phenolic content (TPC) of the fruit peel in the early stages of maturation. In that study, declines to 50.2 mg/g dry weight at harvest maturity stage were indicated. Similarly, Attanayake et al. [17] investigated the changes in TPC, punicalagin α and β, and antioxidant activity of ‘Nimali’ pomegranate peel from the flowering to maturity stage. The recorded gradual declines in TPC from approximately 51 mg GAE/g fresh weight to 31 mg GAE/g fresh weight occurred during the flowering stage to the maturity of the fruit. Li et al. [18] also showed variation in TPC values in sweet GP pomegranate peel that was correlated to different maturities, namely, unripe, half ripe, and ripe fruit. They found that at the unripe maturity stage, the highest TPC was observed at 390 mg GAE/g dry weight compared to when the fruits were ripe at 250 mg GAE/g dry weight. During the maturation and ripening of two Israeli commercial cultivars, namely ‘Wonderful’ and ‘Rosh-Hapered’, TPC and hydrolysable tannins declined significantly [50]. Lowered TPC is assumed to be associated with oxidation reactions catalyzed by polyphenol oxidase, which characterizes these maturity stages [2,50]. Fruit maturation is a biochemically complex process that is physiologically and genetically regulated. It is associated with a halting of new biosynthesis of polyphenols during fruit maturation and phenolic compound involvement in the biosynthesis of compounds with a flavylium ring that drives the accumulation of anthocyanins [2,51]. Such processes are thus involved in causing alterations to the TPC pool as the fruits ripens.

Anthocyanins and other flavonoids upstream the biosynthesis pathway may have independent regulatory mechanisms that are in control despite sharing the same precursors at some point of the biosynthetic pathway. Medlicott et al. [52] studied the changes in peel pigmentation during ripening in mango fruit (*Mangifera indica* var. Tommy Atkins) and recorded a significant decrease in anthocyanin content in the peel during ripening. They suggested that this may be caused by environmental factors during growth and development such as a lack of sunlight and temperature conditions amongst others, which negatively affected the level of phenylalanine ammonia-lyase (PAL) activity, therefore reducing rapid anthocyanin accumulation. Contrary to our findings, in most species, the fruit anthocyanin concentrations increased with ripening/maturation, as their biosynthesis proceeds faster than fruit expansion [53]. This is in agreement with the findings stated by Rivera-López et al. [54], who studied the changes in anthocyanin concentration in the lychee (*Litchi chinensis* Sonn.) pericarp during maturation, where an increase in anthocyanin from 0.1 mg/100 g in the unripe stage of development up to 46 mg/100 g at ripeness or maturity stage was noted. Similarly, in ‘Jonagold’ apple skin, anthocyanin content was very low (almost zero) during the 19th week after full bloom and steadily increased with increase in maturity (25th week after full bloom) to 1.2 mg/g dry weight [55], while, no changes in the contents of the anthocyanins were observed in ‘Amrapali’, ‘Arka Anmol’ and ‘Janardhad Pasand’ mango peels during maturation [56]. Extracts high in anthocyanins are thus sought after in the food and beverage industries as they can improve visual acuity, antioxidant activity, and they also act as chemoprotecting agents [57].

The main goal of blanching is to inactivate enzymes such as polyphenol oxidase (PPO) and peroxidase (POD), responsible for quality and nutritional qualities [20,21,23]. Therefore, understanding the mechanism of degradation of phytochemicals by enzymatic activity and the role of blanching in pomegranate peel could be critical in preservation, extraction, and even the formulation of value-added products from blanched pomegranate peel extracts. Similarly to our findings, Nurhuda et al. [19] observed significant (*p* < 0.05) reductions in PPO and POD activity in ‘Anak Sekolah’ rambutan fruit peel after blanching for 2.5 min and 5 min at 100 °C. Deylami et al. [40], reported that PPO activity in mangosteen pericarp significantly (*p* < 0.05) lowered, however, even after 12 min of blanching, PPO activity remained at 23% and 14% when subjected to blanching temperatures of 90 °C and 100 °C, respectively. They further reported that PPO activity changed during fruit ripening, therefore, it is critical to ascertain the maturity status of the fruit sample tested. Moreover, by applying optimal blanching conditions, it is possible to obtain an acceptable inactivation of enzymes or acceptable low enzyme activity while lessening quality degradation [20,40]. Similarly to our findings, Nurhuda et al. [19] highlighted the importance of pre-treatment of ‘Anak Sekolah’ rambutan peel extracts using hot water blanching at 100 °C for 2.5 min, which demonstrated a significant rise in anthocyanin extraction from 1.00 mg/100 g from untreated peel extracts to 1.39 mg/100 g in blanched peel extracts.

In fruit, most phytochemicals are concentrated in the skin or peel and blanching can induce tissue or cellular disruption/rupture and cause significant phytochemical release from the peel and vacuole/plant cellular compartments, thus increasing the extractability of phytochemicals, however, intense heat of the blanching water (temperature) and prolonged blanching periods (time) may cause over blanching resulting in the loss of phytochemicals into the water medium known as leaching [3,20]. For example, contrary to our findings, Duarte et al. [22] studied the effects of blanching at 90 °C for 1 min, for yellow passion fruit peel (*Passiflora edulis* var. flavicarpa). This investigation determined significantly lower TPC at 3.82 ± 0.06 mg GAE/g DM compared to unblanched peel at 5.62 ± 0.05 mg GAE/g DM. Similarly, Sengkhamparn et al. [23] evaluated the effects of blanching for pitaya (*Hylocereus undatus*) peel, which gave a significantly reduced anthocyanin content after blanching at 95 ± 2 °C for 1 min. The authors stated that blanched pitaya peel dried at 60, and 70 °C had significant reductions of anthocyanin content after blanching at 35.00 mg/g DM and 1.29 mg/g DM, respectively, compared to dried unblanched peel at 38.57 mg/g DM and 5 mg/g DM, respectively. Moreover, Chung et al. [24] observed that significant declines up to 40% were noted in TPC of yam (*Dioscorea alata*) peel blanched at 85 °C for 30 s. The authors attributed this to leaching of water-soluble polyphenols from the peel during blanching. Loss of phytochemicals into the water medium is a critical point to control during water blanching, and the period in which plant material are submersed into the hot water may lead to further losses to quality and nutritional properties of the blanched product [3,20]. Thermal stability of phytochemicals plays a key role in determining the rate of degradation during blanching and could vary even within the same plant source. For example, vitamin C content increased with advancing maturity, however during blanching of peel, a significant (*p* < 0.05) reduction in vitamin C content was noted at unripe and over ripe, while at the ripe harvest stage a significant increase was recorded. Heat stress can cause various changes at cellular and sub-cellular levels, thus, the response of the plant depends on the growth stage, intensity, and duration of exposure to heat stress [58]. In most cases, heat stress such as blanching may cause denaturation or inactivation of enzymes present in the chloroplasts and mitochondria, which are responsible for the degradation of phytochemicals and ascorbic acid concentrations, and for the disruption of membrane integrity, therefore allowing for greater extraction of these valuable phytochemicals [3,19,20,23]. Plants subjected to heat stress may release a number of enzymatic antioxidants such as ascorbate peroxidase and glutathione reductase and non-enzymatic antioxidants such as ascorbic acid or glutathione as a response mechanism against the oxidative stress caused by the heat stress [59,60]. Even though vitamin C contents are heat sensitive and are easily degraded by excessive heat and water [6,61], endogenous ascorbic acid in plant cells plays a key role in plant stress signaling, with the cytoplasm having the largest pool of this metabolite compared with the apoplast. These two pools of ascorbic acid occur at different levels during fruit development, inducing phenotypically plastic responses of plant organs to stress. This may be one reason for different observed responses in terms of the accumulation of vitamin C in relation to the blanching of tissues at different maturity stages [62]. The effect of blanching on quality and nutritional properties such as chemical changes of phytochemicals that may affect the colour and flavour of the blanched product depends on several factors such as the type and size of plant material, thermal stability of different phytochemicals, location of phytochemicals within the plant structure, enzyme activity, and cultivar differences [3,19,20,21,23,25].

Our antioxidant findings are in agreement with those previously described for ‘Nimali’ pomegranate peel [17]. The reduction in total antioxidant activity could be a direct result of the decline in total phenolic content as the fruit matures [2,50]. High total phenolic content has been credited for the high antioxidant activity available in pomegranate peel and various other fruit peels [57,63]. However, we noted a significant (*p* < 0.05) increase in the antioxidant activity of blanched pomegranate peel extracts from all three harvest maturities tested, following the same trend as the improved phytochemical attributes by blanching for all harvest maturities, suggesting higher phytochemical content ascribed to increased extractability in loosened cellulosic structures within the peel as a result of blanching. The stronger antimicrobial activity in blanched peel extracts could be a result of blanching effect on the peel extracts as it causes structural changes in plant tissue, which renders the cell wall more permeable for phytochemical extraction leading to increased bioavailability [3,21,23]. Imposed antioxidant and antibacterial activity in blanched peel extracts is thought to be due to additive and synergistic interactions of complex mixtures of phytochemicals such as hydrolysable tannins and many other bioactives present in the pomegranate peel extracts [3,8,11,12]. During blanching at optimum temperatures of 80 °C for 3 min, release of these compounds may likely occur, thus influencing bacterial activity of ‘Wonderful’ peel extracts. In recent years, many studies have shown that phytochemicals in pomegranate peel may exert antimicrobial activity through different mechanisms of action [3,8,11,12]. For instance, some phytochemicals such as ellagitannins (punicalagin) may act by inhibiting microbial growth by donating or transferring hydroxyl residues to free radicals, consequently quenching these harmful species [11,12]. Some researchers have suggested that ellagitannins in the peel may damage the bacterial membrane and/or even cause cell death by their ability to precipitate proteins [8,64]. However, others have proposed that phytochemicals interfere with certain microbial metabolic processes or gene expression pathways [8,12,61,64].

Ellagitannins (punicalagin) have multiple hydroxyl groups, and the presence of hydroxyl residues allows for radical scavenging, thereby quenching these harmful species (Figure S3) [11,12]. The hydrophilic portion of the tannin (punicalagin) chemical structure interacts with the polar regions of the membrane while the hydrophobic portion is submerged in a non-polar inner segment of the bacterial membrane, inducing membrane instability and ultimately affecting the transport of substrates into the cell [8,12,64]. Therefore, chemical features of these compounds control the strong biological activity such as antioxidant, antimicrobial, anticancer, and anti-inflammatory activities linked to pomegranates [3,8,11,12,13]. Li et al. [18] reported variation in four polyphenols of pomegranate peel extracts namely punicalagin α and β, punicalin α and β, ellagic acid and gallic acid ranging at 61.75 (‘Sweet red peel’) to 125.23 mg/g D.M (‘Sour Yunnan red peel’), 1.68 (‘Sour red peel’) to 3.91 mg/g D.M (‘Sweet red peel’), 2.68 (‘Sweet Tai-mountain red peel’) to 7.07 mg/g D.M (‘Sweet red peel’), 0.18 (‘Sweet Tai-mountain red peel’) to 0.41 mg/g D.M (‘Sweet red peel’), respectively. Fawole et al. [8] reported that ellagic acid was the most abundant, ranging from 46.87 µg/mL in ‘Ruby’ to 209.44 µg/mL in ‘Ganesh’ methanolic peel extracts. Khalil et al. [65] observed that punicalagin was the most predominant elligatannin at 118.6 mg/g D.M, 110.0 mg/g D.M, and 88.7 mg/g D.M for ‘Kandhari’, ‘Desi’ and ‘Badana’ pomegranate peel extracts, respectively. Similarly, John et al. [41] reported higher α and β punicalins at 0.8 mg/g and 1.6 mg/g, punicalagins (~38.6–50.3 mg/g) and ellagic acid (~2.8–3.2 mg/g) in lyophilized peel. The phenolic contents can be influenced by processing methods such as drying, extraction, and the type of solvent used [3]. Moreover, concentrations of polyphenols can also be affected by other factors such as the cultivar and harvest maturity of the pomegranate fruit peel [3,13,15].

The characterization and quantification of polyphenol compounds in blanched ‘Wonderful’ pomegranate peel samples from three harvest maturities (unripe, ripe, and over ripe) showed that some of the polyphenols presented in the study have strong antioxidant potential. Hydrobenzoic acids and its derivatives, various flavonoids and ellagitannins such as α-punicalagin, β-punicalagin, and punicalin α and β are regarded as potential compounds showing considerable free radical scavenging potential [8,18,42]. Furthermore, blanching at 80 °C for 3 min has been reported in this study as being effective in increasing the extractability of polyphenols by lowering enzymatic (PPO and POD) activity in the peel extracts in all harvest maturities of ‘Wonderful’ pomegranate tested. Additionally, blanching assists in reducing the prolonged drying time of the peel, therefore preserving the integrity of the compounds during thermal processing. Thus, blanching should be considered as a novel pre-treatment step in pomegranate peel during processing for value-addition.

## 5. Conclusions

Higher extraction of key metabolites at significantly higher concentrations due to lowered or complete inactivation of enzymes (PPO and POD) responsible for phytochemical degradation was achieved through blanching. This led to stronger antioxidant and antibacterial activities on all blanched peel extracts compared to unblanched peel extracts regardless of the harvest maturity stage tested. In addition, the highest phytochemical and antioxidant activity was recorded in unripe peel extracts and changes in activity were generally reduced with increasing harvest maturity stage. This lends support to the idea that blanching may be used as a novel pre-treatment step prior to processing of pomegranate peel to enhance the recovery of phytochemicals. Depending on the need of the processor or product manufacturer in the industry, applying blanching treatment to unripe pomegranate peel extracts of ‘Wonderful’ may increase extractability of key metabolites such as flavonoids. On the other hand, blanching over ripe peel extracts is linked to higher extraction of punicalin α and β, α punicalagin, and gallic acid. All polyphenols in both blanched peel extracts are linked to high antioxidant potential, which has potential utility in food, cosmetic, and pharmacological industries. However, all harvest maturities showed higher polyphenol concentrations compared to pomegranate peel studies reported in literature. Therefore, pomegranate peel extracts of ‘Wonderful’, regardless of harvest maturity stage, could be used for value-addition. Ellagitannins such as α-punicalagin, β-punicalagin, and punicalin α and β; flavanols (catechin and epicatechin) and hydroxybenzoic acid (gallic acid) were the most affected by the blanching treatment. The presence of these antioxidant compounds indicates that pomegranate peel waste can be a good source of polyphenols and antioxidant potential. Blanching pre-treatment is simple to use and inexpensive and should be considered as a pre-treatment step during processing for value addition. However, blanching effects may depend on the harvest maturity, cultivar type and various other factors, which include processing methods utilized. In our study, higher extractability of polyphenols and antioxidant activity in unripe peel extracts compared to other maturities was evident. To our knowledge, we are the first to successfully use hot water blanching in pomegranate peel as a method for antioxidant extraction and report on its effects on different harvest maturities in pomegranate peel, which is an opportunity for value addition at different harvest stages. This paper thus adds new information that can easily be adopted by any processor or product manufacturer in the industry to obtain high phytochemical yields, in a manner that is environmentally friendly, safe, and cost effective using pomegranate peel. Hot water blanching is a simple and yet cost-effective pre-treatment step that enables the release of these phytochemicals for value addition. Further work involving the optimization of blanching temperature and time for ‘Wonderful’ pomegranate peel extracts and toxicity effects are necessary. In addition, biochemical and genetic events that control metabolite pools (including ascorbic acid) may further be resolved using transcriptomic and proteomic-based studies to provide insights into the molecular mechanisms at play during blanching at different harvest stages of pomegranate peels.

## Figures and Tables

**Table 1:** Extraction yield, bioactive compounds, enzyme activity and antioxidant activity of ethanol (70% (*v*/*v*)) extracts from three harvest maturities (unripe, ripe, and over ripe) of ‘Wonderful’ pomegranate peel blanched (80 °C for 3 min).

Harvest Maturity	Treatment	Extract Yield (%)	TPC	TTC	TFC	TAC	Vit C	DPPH	ABTS	FRAP	PPO	POD
Unripe	Unblanched	28.1 ± 0.6 ^c^	12.1 ± 0.1 ^b^	0.9 ± 0.1 ^b^	0.8 ± 0.1 ^d^	0.09 ± 0.00 ^b^	38.3 ± 0.3 ^e^	279.1 ± 5.6 ^c^	721.8 ± 1.2 ^c^	541.1 ± 89.2 ^b^	0.5 ± 0.0 ^b^	3.8 ± 0.0 ^b^
	Blanched	31.3 ± 0.6 ^ab^	14.0 ± 0.0 ^a^	1.0 ± 0.1 ^a^	1.1 ± 0.1 ^c^	0.10 ± 0.00 ^a^	31.0 ± 0.1 ^f^	359.2 ± 0.0 ^a^	912.2 ± 0.0 ^a^	802.5 ± 4.3 ^a^	0.2 ± 0.1 ^e^	1.5 ± 0.0 ^d^
Ripe	Unblanched	29.5 ± 0.8 ^bc^	10.6 ± 0.2 ^d^	0.8 ± 0.0 ^c^	1.2 ± 0.0 ^c^	0.07 ± 0.00 ^e^	45.2 ± 0.1 ^d^	243.9± 2.4 ^d^	718.8 ± 2.4 ^c^	478.0 ± 74.0 ^c^	0.4 ± 0.0 ^c^	3.0 ± 0.0 ^c^
	Blanched	33.8 ± 1.4 ^a^	12.2 ± 0.1 ^b^	1.1 ± 0.1 ^a^	1.5 ± 0.0 ^b^	0.08 ± 0.00 ^c^	53.9 ± 0.5 ^c^	319.2 ± 4.2 ^b^	778.8 ± 2.4 ^b^	525.3 ± 15.8 ^b^	0.3 ± 0.0 ^d^	1.5 ± 0.0 ^d^
Over Ripe	Unblanched	26.8 ± 1.0 ^d^	9.5 ± 0.0 ^e^	0.6 ± 0.0 ^d^	1.4± 0.1 ^b^	0.07 ± 0.00 ^e^	77.7 ± 0.2 ^a^	208.8 ± 2.4 ^e^	612.2 ± 0.0 ^d^	370.1 ± 3.6 ^d^	0.6 ± 0.0 ^a^	4.5 ± 0.0 ^a^
	Blanched	30.9 ± 0.3 ^b^	11.6 ± 0.0 ^c^	0.9 ± 0.1 ^b^	1.8 ± 0.0 ^a^	0.08 ± 0.00 ^d^	70.5 ± 0.1 ^b^	316.7 ± 2.4 ^b^	614.7 ± 0.6 ^d^	447.7 ± 98.8 ^c^	0.5 ± 0.0 ^b^	1.5 ± 0.0 ^d^

Value are means ± SE of triplicate (*n* = 3) determinations. Different letter(s) ^(a–f)^ in each column indicate statistical significance (*p* < 0.05) differences according to Duncan’s multiple range test. Unripe, ripe, and over-ripe stands for two weeks before commercial harvest, at commercial harvest, and two weeks after commercial harvest, respectively. Extract yield = % per g DM TPC = total phenolic content (mg GAE/g DM), TTC = total tannin content (mg GAE/g DM), TFC = total flavonoid content (mg CE per g peel extracts), TAC = total anthocyanin content (mg Cy3dE/g DM), Vit C = vitamin C (ug of AAE/g DM), DPPH = 2,2 diphenyl-1-picryl hydrazyl assay (µmol Trolox/g DM), FRAP = ferric reducing antioxidant power assay (µmol Trolox/g DM), 2,2-azino-bis(3-ethylbenzothiazoline-6-sulphonic acid assay (µmol Trolox/g DM), GAE = gallic acid equivalent, CE = catechin equivalent, Cy3De = cyanidin-3-glucoside equivalent, AAE = ascorbic acid equivalent, PPO = polyphenol oxidase (U/g FW), POD = peroxidase (U/g FW) unit per gram of fresh weigh.

**Table 2:** Individual phenolic and flavonoid concentration from three harvest maturities (unripe, ripe, and over ripe) of ‘Wonderful’ pomegranate peel blanched at 80 °C for 3 min.

Harvest Maturity/Treatment						
Bioactive Compounds	Unripe		Ripe		Over Ripe	
	Unblanched	Blanched	Unblanched	Blanched	Unblanched	Blanched
Punicalin α and β	185 ± 3 ^d^	414 ± 6 ^a^	228 ± 5 ^c^	342 ± 9 ^b^	237 ± 4 ^c^	436 ± 16 ^a^
α-Punicalagin	598 ± 13 ^b^	618 ± 12 ^b^	562 ± 13 ^c^	610 ± 11 ^b^	523 ± 10 ^d^	659 ± 19 ^a^
β-Punicalagin	696 ± 18 ^a^	678 ± 18 ^b^	592 ± 33 ^d^	644 ± 35 ^c^	514 ± 19 ^d^	641 ± 38 ^b^
Ellagic acid	308 ± 18 ^bc^	279 ± 16 ^c^	324 ± 22 ^b^	297 ± 34 ^bc^	306 ± 17 ^bc^	363 ± 36 ^a^
Catechin	99 ± 2 ^b^	151 ± 6 ^a^	55 ± 4 ^d^	84 ± 4 ^c^	93 ± 3 ^c^	80 ± 3 ^c^
Epicatechin	12 ± 1 ^c^	227 ± 1 ^a^	123 ± 1 ^c^	10 ± 1 ^c^	20 ± 1 ^a^	16 ± 2 ^b^
Gallic acid	139 ± 2 ^c^	85 ± 1 ^d^	13 ± 4 ^c^	163 ± 4 ^b^	165 ± 6 ^b^	200 ± 9 ^a^

Mean in column with different letter(s) ^(a–d)^ differ significantly (*p* < 0.05) according to Duncan’s multiple range test. (*n* = 5). Means ± SE presented. Unripe, ripe, and over ripe represent two weeks before commercial harvest, commercial harvest, and two weeks after commercial harvest, respectively.

**Table 3:** Antibacterial activity (MIC, µg/mL) of ethanol (70% (*v*/*v*)) extracts from blanched (80 °C, 3 min) ‘Wonderful’ pomegranate peel extracts at 3 different harvest maturities (unripe, ripe, and over ripe).

Harvest Maturity	Treatment	Gram Negative		Gram Positive	
		*Escherichia coli*	*Klebsiella pneumonia*	*Staphylococcus aureus*	*Bacillus subtilis*
Unripe	Unblanched	310	310	310	310
	Blanched	160	160	160	160
Ripe	Unblanched	310	310	310	310
	Blanched	160	160	160	160
Over Ripe	Unblanched	310	310	310	310
	Blanched	160	160	160	160
Streptomycin (µg/mL)		1.6	1.6	0.8	1.6
Solvent Control (70% Ethanol)		-	-	-	-

- Denotes not inhibited.

**Table 4:** Pearson’s correlation matrix between chemical indices measured from blanched (80 °C for 3 min) ‘Wonderful’ pomegranate peel from three harvest maturities.

	EY	TPC	TTC	TFC	TAC	Vit C	DPPH	FRAP	PPO	POD	Pα&β	αPun	βPun	EA	Cat	GA	*E. c*	*K. p*	*S.a*	*B. s*
EY	**1**																			
TPC	0.61	**1**																		
TTC	**0.83**	**0.88**	**1**																	
TFC	0.29	**0.92**	0.70	**1**																
TAC	0.39	−0.23	−0.08	−0.40	**1**															
Vit C	−0.24	−0.74	−0.61	−0.70	0.74	**1**														
DPPH	0.77	**0.93**	**0.88**	0.80	0.11	−0.50	**1**													
FRAP	0.24	0.27	−0.18	−0.39	**0.97**	0.75	0.07	**1**												
PPO	−0.55	0.02	−0.16	0.26	**−0.90**	−0.46	−0.31	**−0.90**	**1**											
POD	**−0.86**	−0.52	−0.57	−0.24	−0.64	0.01	−0.74	−0.54	0.72	**1**										
Pα&β	0.67	0.54	0.48	0.39	0.65	0.07	0.78	0.62	−0.69	**−0.93**	**1**									
αPun	0.65	0.69	0.71	0.56	0.34	−0.22	0.85	0.38	−0.61	−0.65	0.74	**1**								
βPun	0.48	**0.87**	**0.84**	**0.83**	−0.33	−0.73	0.80	−0.30	0.01	−0.24	0.29	0.76	**1**							
EA	−0.09	−0.41	−0.32	−0.45	0.59	0.54	−0.17	0.74	−0.72	−0.07	0.17	0.34	−0.13	**1**						
Cat	0.09	0.71	0.39	**0.85**	−0.31	−0.45	0.58	−0.32	0.39	−0.21	0.39	0.22	0.42	−0.64	**1**					
GA	−0.01	−0.54	−0.28	−0.57	0.73	**0.84**	−0.27	0.76	−0.64	−0.04	0.09	0.16	−0.31	0.75	−0.60	**1**				
*E. c*	**−0.85**	−0.67	−0.71	−0.47	−0.56	0.06	**−0.87**	−0.47	0.62	**0.93**	**−0.94**	−0.79	−0.45	−0.01	−0.39	−0.08	**1**			
*K. p*	**−0.85**	−0.67	−0.71	−0.47	−0.56	0.06	**−0.87**	−0.47	0.62	**0.93**	**−0.94**	−0.79	−0.45	−0.01	−0.39	−0.08	**1.00**	**1**		
*S. a*	**−0.85**	−0.67	−0.71	−0.47	−0.56	0.06	**−0.87**	−0.47	0.62	**0.93**	**−0.94**	−0.79	−0.45	−0.01	−0.39	−0.08	**1.00**	**1.00**	**1**	
*B. s*	**−0.85**	−0.67	−0.71	−0.47	−0.56	0.06	**−0.87**	−0.47	0.62	**0.93**	**−0.94**	−0.79	−0.45	−0.01	−0.39	−0.08	**1.00**	**1.00**	**1.00**	1

Values in bold are different from 0 with a significance level alpha = 0.05. Abbreviations: Extract yield (EY), total phenolic content (TPC), total tannin content (TTC), total flavonoid content (TFC), total anthocyanin content (TAC), vitamin C (Vit C), 2,2-diphenyl-1-picryl hydrazyl (DPPH) free radical scavenging assay, ferric ion reducing antioxidant power (FRAP), polyphenol oxidase (PPO), peroxidase (POD), punicalin α&β (Pα&β), α-punicalagin (αPC), β-punicalagin (βPC), ellagic acid (EA), catechin (Cat), epicatechin (Epi), gallic acid (GA), *E. coli* (*E.c*), *K. pneumonia (K.p), S. aureus (S.a), B. subtilis (B.s)*.

**Table 5:** Individual phenolic and flavonoid concentration in blanched pomegranate peel (‘Wonderful’ unripe, ripe, and over ripe harvest maturity).

No.	Tentative ID	Retention Time	M-H	MS^E^ Fragment Ions	UV Max	Elemental Formula	References
**Phenolic acids**							
	**Hydroxybenzoic acids**						
1	Gallic acid *	4.72	169.0146	**169.014**, 125.025, 124.017	270,259	C_7_H_5_O_5_	Standard
2	Ellagic acid *	14.24	300.9969	**213.597**, 137.983, 49.474	254,364	C_14_H_5_O_8_	Standard
3	Ellagic acid hexoside	11.06	463.0539	**463.053**, 300.989, 165.021,114.6995	-	C_20_H_15_O_13_	[41]
4	Ellagic acid pentoside	13.59	433.0307	**433.038**, 303.7598, 300.995, 299.997, 201.556, 126.237	254,361	C_19_H_13_O_12_	[41,42]
	**Organic acids**						
5	Citric acid *	2.52	191.0198	**191.0198**, 173.008, 111.008,87.008, 67.017	-	C_5_H_7_O_7_	Standard
**Flavonoids**							
	**Flavanols**						
6	(+)-Catechin *	8.31	289.0733	**289.071**, 245.082, 203.072, 109.028		C_15_H_13_O_6_	Standard
							
7	(−)-Epicatechin *	10.19	289.0733	**289.071**, 245.082, 203.072, 109.028		C_15_H_13_O_6_	Standard
	**Flavones**						
8	Apigenin-7-O-glucoside **Flavonols**	11.07	431.1906	**161.041**, 153.091	253, 361	C_20_H_20_O_10_	[42]
9	Kaempferol-3-O-rutinoside	15.95	593.1494	**593.142**, 523.421, 440.063, 316.023, 300.998, 285.033, 211.911, 125.025, 101.031, 80.779	275,360	C_27_H_29_O_15_	[43]
10	Punicalin α and β *	4.985	781.0506	**781.022**, 779.000,783.055, 784.065	270, 259	C_34_H_21_O_22_	Std
11	α Punicalagin *	6.46	1083.0547	**1083.060**, 781.035, 600.986, 541.027, 300.997	258,378	C_48_H_27_O_30_	Std
12	β Punicalagin *	7.69	1083.0558	**1083.060**, 781.035, 600.986, 541.027, 300.997	258,378	C_48_H_27_O_30_	Std
13	Punicalagin isomer	5.305	1083.0547	**1083.060**, 781.035, 600.986, 541.027, 300.997	258,378	C_48_H_27_O_30_	[41,42]
14	Punicalagin derivative	6.44	541.0341	**541.027**, 300.997	258,378	-	[44]
15	Galloyl- hexahydroxydiphenol-dehydrohexahydroxydiphenol-hexoside	6.83	951.0479	**951.042**, 907.087, 820.125, 783.062, 300.997, 275.021, 249.035, 102.241	255, 230	C_52_ H_23_O_19_	[42,44]
16	Galloyl- hexahydroxydiphenol -dehydrohexahydroxydiphenol-hexoside (Granatin B)	12.43	951.071	**951.044**, 933.079, 915.9995, 763.081, 614.803, 464.044, 341.011, 302.006, 300.999, 273.002, 169.013, 1233.011	273	C_52_H_23_O_19_	[42,44]
17	Granatin A	8.34	799.0454	**799.0454**, 800.0641, 802.0781		C_34_H_23_O_23_	[45,46]
18	Digalloyl- hexa hydroxydiphenol-gluconic acid (punigluconin)	8.60	801.0732	**801.073**, 781.3245	-	C_41_H_21_O_18_	[46]
19	Digalloyl- hexahydroxydiphenol-hexoside (Pedunculagin II))	9.37	785.0775	**785.021**, 781.795, 635.099, 483.122, 419.050, 345.086, 301.995, 300.9982, 275.032, 165.021, 125.132	264,370	C_34_H_25_O_22_	[41,42,45]
20	Galloyl-hexahydroxydiphenol-hexoside	9.76	633.0686	**633.075**, 597.082, 464.051, 301.9998, 275.0219, 125.024	260,360	C_27_H_22_O_18_	[44,47]
21	Digalloyl-gallagyl-hexoside	9.99	1085.0889	**933.081**, 783.088, 633.0696, 540.536, 450.989, 301.996, 300.997, 275.014,	257,370	C_48_H_29_O_30_	[41,47]
22	Bis-hexahydroxydiphenol-hexoside (pedunculagin I)	8.83	783.0746	**783.567**, 635.087, 541.022, 453.103, 392.036, 291.016, 203.0396	260,370	C_34_H_23_O_22_	[41,44]
**Unknown**							
23	Unknown a	1.58	217.0461	**217.042**, 191.0211, 173.006	-	C_12_H_9_O_4_	-
24	Unknown b	2.37	353.0731	**353.0742**, 191.0211, 173.0062	-	C_12_H_17_O_12_	-
25	Unknown c	12.81	539.2146	**541.038**, 492.1699, 405.014, 328.021, 302.001, 300.998, 222.005, 169.011, 52.256	254,230	C_26_H_35_O_12_	-

* Confirmed using a pure chemical standard. Literature sources and *standards were used to corroborate existing observations. MS^E^ fragments in bold type face refer to the base peak (the highest peak) [48].

## Data Availability

Data is contained within the article.

## Supplementary Materials

The following are available online at https://www.mdpi.com/article/10.3390/antiox10071119/s1, Figure S1: Examples of chromatograms of ‘Wonderful’ pomegranate peel extracts obtained from three harvest maturities (unripe, ripe, and over ripe) and blanched at 80 °C for 3 min. Compound numbers correspond to Table 5. Figure S2: Dendrogram representing different sub-clusters according to the similarities of their concentration patterns in ‘Wonderful’ pomegranate peel extracts obtained from three harvest maturities (unripe, ripe, and over ripe) and blanched at 80 °C for 3 min. Figure S3: Chemical structures of some phenolic acids, flavonoids, and ellagitannins identified in three different harvest maturities (unripe, ripe, and over ripe) of blanched ‘Wonderful’ pomegranate peel extracts.
