# Supplementary material for: Blanching Pre-Treatment Promotes High Yields, Bioactive Compounds, Antioxidants, Enzyme Inactivation and Antibacterial Activity of ‘Wonderful’ Pomegranate Peel Extracts at Three Different Harvest Maturities
Source: Antioxidants (Basel). 2021 Jul 13;10(7):1119. doi: 10.3390/antiox10071119 (PMC8301009; doi:10.3390/antiox10071119)
Supplement: Supplementary file 1 [file antioxidants-10-01119-s001.zip › antioxidants-1245079-supplementary.pdf]

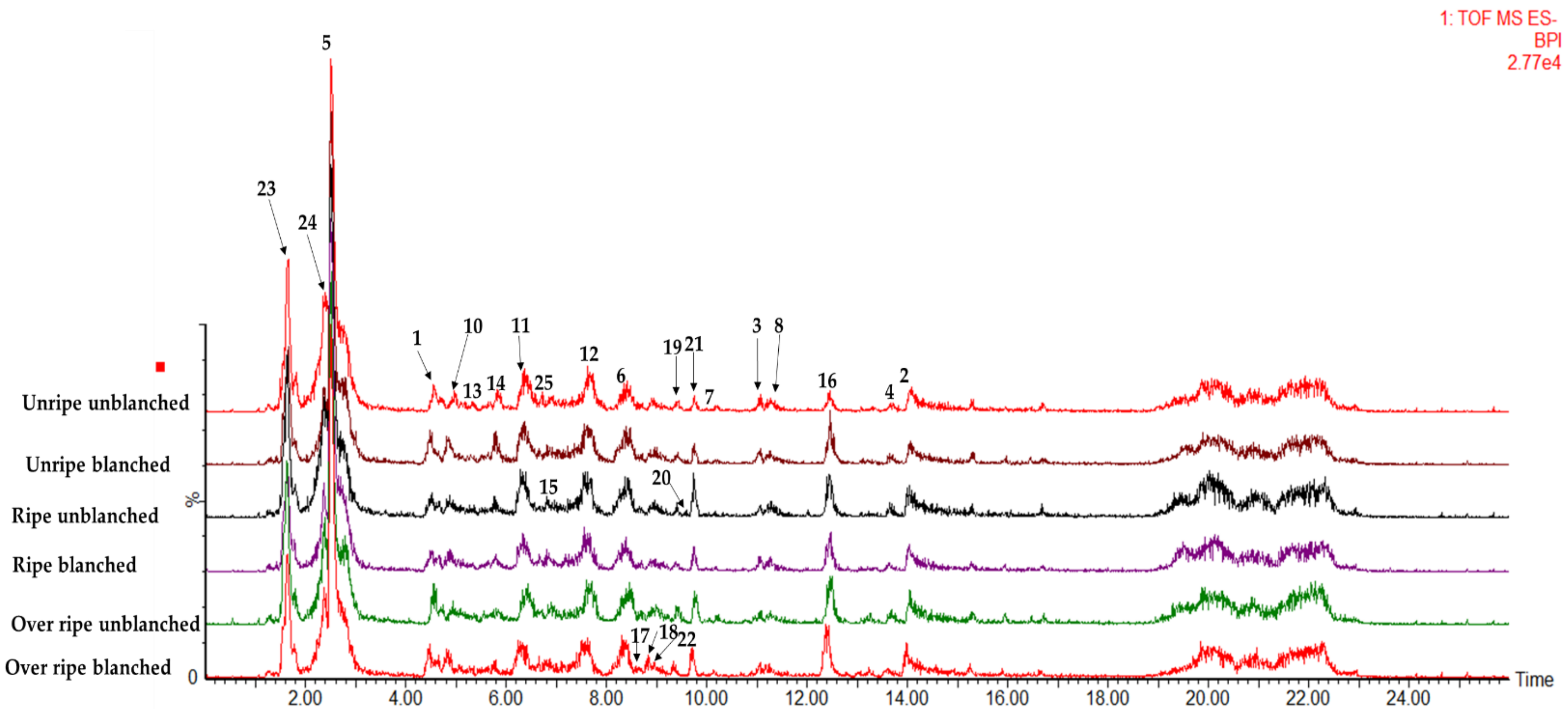

Figure S1 Examples of chromatograms of 'Wonderful' pomegranate peel extracts obtained from three harvest maturities (unripe, ripe, and over ripe) and blanched at 80 °C for 3 min. Compound numbers correspond to Table 5.

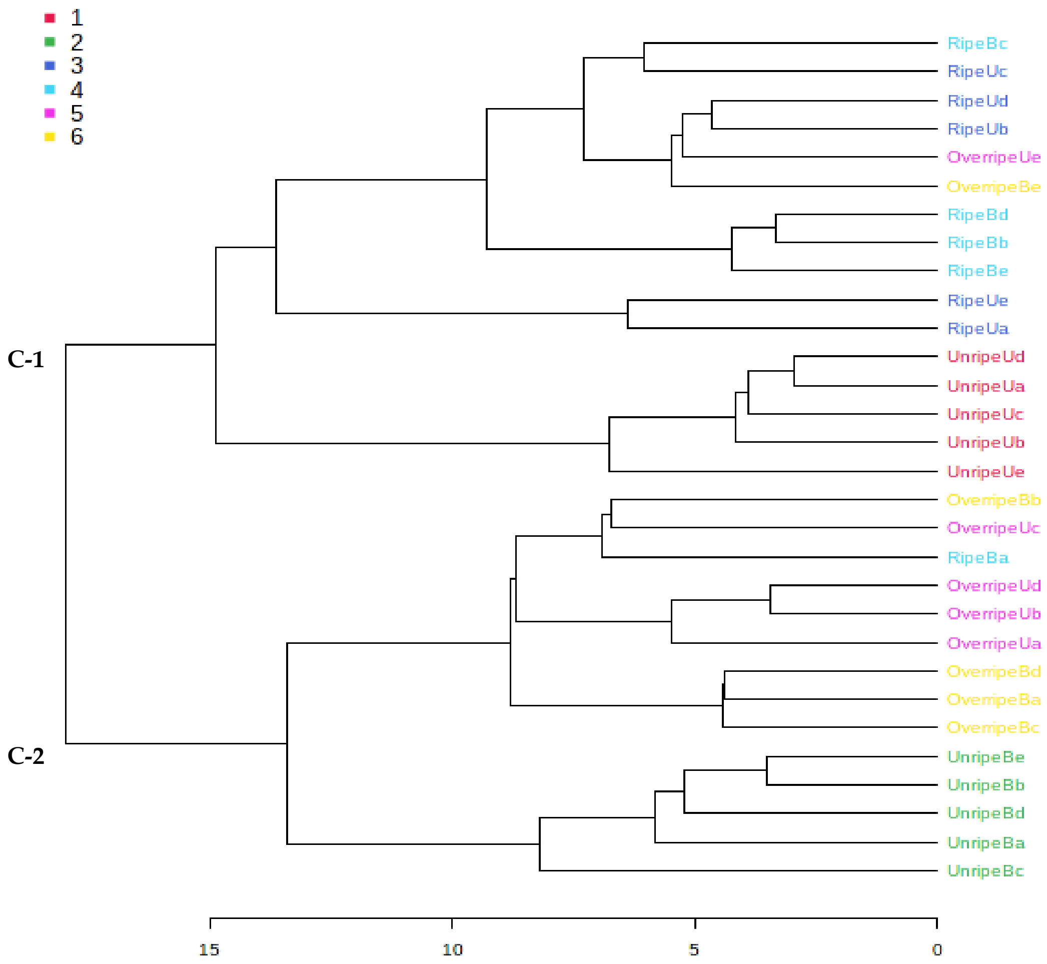

Figure S2 Dendrogram representing different sub-clusters according to the similarities of their concentration patterns in 'Wonderful' pomegranate peel extracts obtained from three harvest maturities (unripe, ripe, and over ripe) and blanched at 80 °C for 3 min.

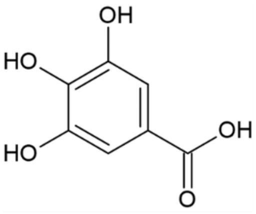

Gallic acid (1)

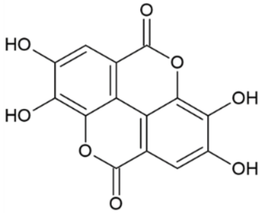

Ellagic acid (2)

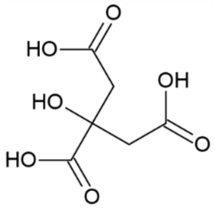

Citric acid (5)

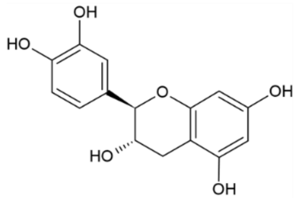

Catechin (6,7)

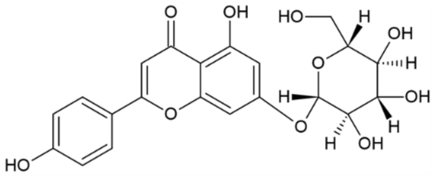

Apigenin-7-O-glucoside (8)

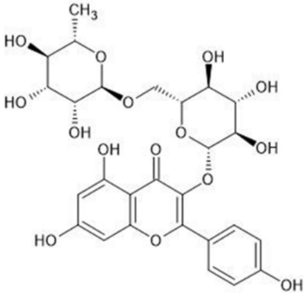

Kaempferol-3-O-rutinoside (9)

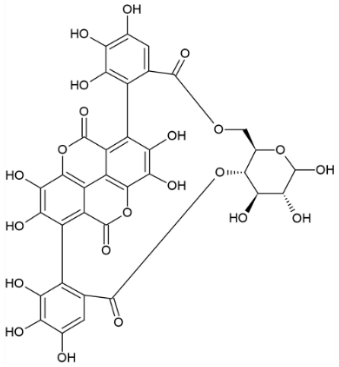

Punicalin α and β (10)

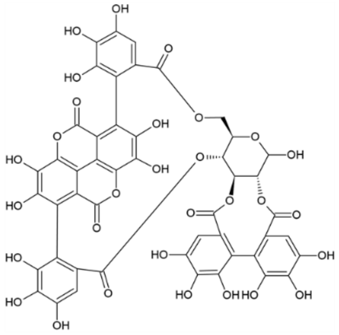

Punicalagin α and β (11, 12)

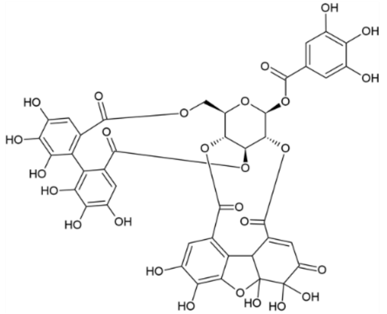

Galloyl-HHDP-DHHDP-hexoside (granatin B) (15,16)

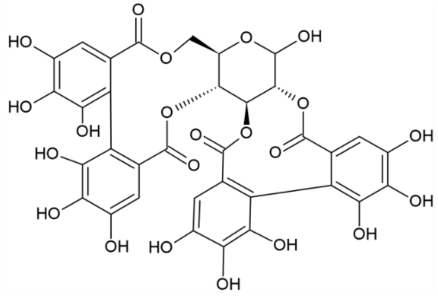

Bis-HHDP-hexoside (Pedunculagin I) (22)

Figure S3 Chemical structures of some phenolic acids, flavonoids, and ellagitannins identified in three different harvest maturities (unripe, ripe, and over ripe) of blanched 'Wonderful' pomegranate peel extracts.
